# Drought Tolerance and Application of Marker-Assisted Selection in Sorghum

**DOI:** 10.3390/biology10121249

**Published:** 2021-11-30

**Authors:** Andekelile Mwamahonje, John Saviour Yaw Eleblu, Kwadwo Ofori, Santosh Deshpande, Tileye Feyissa, Pangirayi Tongoona

**Affiliations:** 1 West Africa Centre for Crop Improvement (WACCI), College of Basic and Applied Sciences, University of Ghana, Accra 00233, Ghana; jeleblu@wacci.ug.edu.gh (J.S.Y.E.); kofori@wacci.ug.edu.gh (K.O.); ptongoona@wacci.ug.edu.gh (P.T.); 2 Makutupora Centre, Tanzania Agricultural Research Institute (TARI), P.O. Box 1676, Dodoma 41026, Tanzania; 3 International Crops Research Institute for the Semi-Arid Tropics, Patancheru 502324, India; s.deshpande@cgiar.org; 4 Institute of Biotechnology, Addis Ababa University, P.O. Box 1176, Addis Ababa 1000, Ethiopia; tileye.feyissa@aau.edu.et

**Keywords:** drought tolerance, marker-assisted selection, post-flowering, QTL mapping, sorghum, stay-green

## Abstract

**Simple Summary:**

Sorghum is a climate-resilient crop grown in limited rainfall areas globally. However, climate change has increased temperature and shortened rainfall durations, which has constrained crop yield. We reviewed mechanisms of drought tolerance and application of marker-assisted selection in sorghum. Marker-assisted selection uses DNA molecular markers to map quantitative trait loci (QTL) associated with stay-green. *Stg1, Stg2, Stg3, Stg4, Stg3A,* and *Stg3B* QTLs associated with stay-green and high yield, have been mapped in sorghum. These QTLs are used for introgression into the senescent sorghum varieties through marker-assisted backcrossing.

**Abstract:**

Sorghum is an important staple food crop in drought prone areas of Sub-Saharan Africa, which is characterized by erratic rainfall with poor distribution. Sorghum is a drought-tolerant crop by nature with reasonable yield compared to other cereal crops, but such abiotic stress adversely affects the productivity. Some sorghum varieties maintain green functional leaves under post-anthesis drought stress referred to as stay-green, which makes it an important crop for food and nutritional security. Notwithstanding, it is difficult to maintain consistency of tolerance over time due to climate change, which is caused by human activities. Drought in sorghum is addressed by several approaches, for instance, breeding drought-tolerant sorghum using conventional and molecular technologies. The challenge with conventional methods is that they depend on phenotyping stay-green, which is complex in sorghum, as it is constituted by multiple genes and environmental effects. Marker assisted selection, which involves the use of DNA molecular markers to map QTL associated with stay-green, has been useful to supplement stay-green improvement in sorghum. It involves QTL mapping associated with the stay-green trait for introgression into the senescent sorghum varieties through marker-assisted backcrossing by comparing with phenotypic field data. Therefore, this review discusses mechanisms of drought tolerance in sorghum focusing on physiological, morphological, and biochemical traits. In addition, the review discusses the application of marker-assisted selection techniques, including marker-assisted backcrossing, QTL mapping, and QTL pyramiding for addressing post-flowering drought in sorghum.

## 1. Introduction

A drought is a condition when soil moisture fails to support plant growth due to low normal precipitation. Low moisture and groundwater in the soil is caused by insufficient rainfall, which results in crop damage and poor yield [1]. Meteorological drought is a drought that is based on the degree of dryness for the specific period of time; this type of drought depends on geographical regions. There are cereal crops that are tolerant to drought, for instance, millets and sorghum, which need further studies to understand their mechanisms of survival. Sorghum adapts well in marginal lands with low rainfall and fragile soils with low fertility where other cereal crops usually fail [2,3]. The crop is a C_4_ plant characterized by high efficiency in hot, dry climates and makes a great deal of energy to exploit water and nutrients from the soil. Nevertheless, droughts caused by gradual climate change occurring globally affect the crop performance because of insufficient moisture to support plant growth to maturity. Impact of climate change projects that the yield will decrease by 19–20% on cereal crops except sorghum and millets, for which yield will decrease less than other cereals from 2040–2069. Therefore, there is a need to focus on crops that will be impacted less by climate change, such as sorghum and millets [4].

Efforts have been made to study the mechanism of drought tolerance in sorghum concentrating on three sections, including physiological, morphological, and biochemical mechanisms [5,6,7,8,9,10,11]. However, mechanisms that make sorghum a drought-tolerant and stable grain yield crop have not been sufficiently elucidated. Multi-approach strategies to understand the mechanism of the crop tolerance are paramount to exploit traits that are important for further improvement to address drought, which is projected to increase because of climate change [12]. Climate change is caused by increase in human activities, for instance, agriculture, pastoralism, industrialization, transportation, and constructions of buildings because of population increase, which poses danger of ozone layer destruction, thus increasing drought on crops [13]. Drought affects physiological, morphological, and biochemical plant features; nevertheless, plants have mechanisms to adapt to the constraints [14,15]. Plant age, nutrient deficiency, and diseases are traits to consider when phenotyping for drought tolerance in sorghum [16]. 

Molecular marker studies in response to deploy drought tolerance have supplemented the information that contributes to tolerance in sorghum, though further studies are in progress to innovate modern technology. Marker-assisted selection is among the tools that are used for mapping QTLs conferring drought tolerance in sorghum. For instance, molecular markers with known QTLs for drought tolerance have been useful for backcrossing into varieties with early leaf senescence [17]. The donor parent lines with the trait of drought tolerance enhance sorghum improvement for tolerance stability [18]. Use of donor parents with good heritability and combining ability for drought tolerance-related traits are important for development of hybrids [19]. The breeding of sorghum varieties for drought tolerance, disease resistance, and improved yield increases the production and the productivity per unit area. This is achieved by application of conventional and molecular breeding and biotechnology tools to shorten the breeding cycles [20]. Despite the potential to tolerate many types of stresses, sorghum is affected by post-flowering drought, which affects grain filling [21,22,23]. Sub-seasonal rainfalls, which are projected to increase in Africa, will constrain sorghum production [24]. Therefore, the paper aims to review the mechanisms of drought tolerance in sorghum and the application of marker-assisted selection for enhancing drought tolerance through sorghum breeding programs.

### Impact of Drought in Sorghum

**Figure 1:**
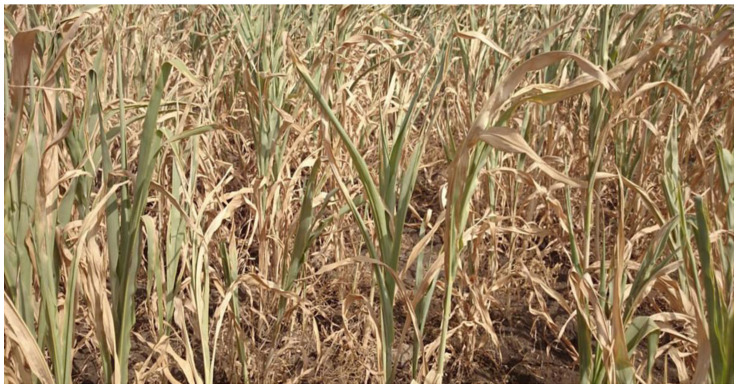
Impacts of drought stress in sorghum in Ethiopia, Photos—January 2016.

Drought affects nutrients and water uptake from the soil. It affects the process of photosynthesis, respiration, and dry matter partitioning in the plants depending on the type of drought [25,26]. Drought affects sorghum plants differently at physiological growth stages, for instance, seedling, pre-flowering, and post-flowering stages, which contribute to the final yield. Pre-flowering and post-flowering drought reduce grain yield [7,27]. Drought stress at the vegetative stage in sorghum causes yield losses of 50–60%. However, water stress at post-flowering growth stage causes yield losses of 87–100% since at this stage, plants need plenty of water for grain filling [28]. It should be noted that the degree of drought impact in sorghum changes from one environment to another. In addition, the ability to maintain leaf greenness differs from one variety to another; nonetheless, retaining of leaf greenness is positively correlated with grain yield [7]. Sorghum, as an important crop to climate resilience, is suitable to smallholder farmers who cannot afford irrigation to sustain food security in Africa and Asia [29]. Climate change has caused unpredictable weather conditions, especially in semi-arid areas where the duration of dry spells has been increasing, which has caused crop wilting, leading to food insecurity. Figure 1 shows the impact of drought stress on sorghum plants in Ethiopia.

## 2. Mechanisms of Drought Tolerance in Sorghum

Drought tolerance is the ability of plants to withstand drought stress while maintaining appropriate physiological activities to stabilize and protect cellular and metabolic integrity at tissue and cellular level [30,31]. It is a complex trait associated with the interactions of environmental factors and genetic differences that allows cell survival with less metabolic activity leading to low yield of plants [32]. Sorghum is affected by drought in early growth stages and pre-flowering and post-flowering growth stages [33]. Drought occurring during the early growth stage weakens growth of sorghum plants due to limited water and nutrients absorption from the soil. Pre-flowering drought is common when plants are at vegetative growth stage to pre-flowering; it affects physiological and morphological plant growth [34]. However, plants have the ability to resume normal growth regenerates after a period of drought. Drought after flowering has a negative effect on the filling of cereals, so it causes a significance loss of crops because at this stage, plants absolutely need an adequate water content in the soil [35]. In addition, plants have the ability to cope with drought stress, including physiological, morphological, and biochemical mechanisms. Physiological mechanism involves the adjustment in photosynthesis, chlorophyll content, stomatal conductance, and transpiration rate. The plants reduce leaf water potential and regulate osmotic potential while keeping high chlorophyll content for photosynthesis to support plant growth. Useful morphological features include adequate plant height, roots development, biomass production, and leaf arrangement to allow plants to adapt during water scarcity, while a biochemical mechanism involves vital processes taking place in plants during drought. Therefore, physiological, morphological, and biochemical mechanisms play the major role in coping with drought stress in sorghum, as described in details below.

### 2.1. Physiological Mechanisms

Sorghum adapts drought stress due to regulated physiological processes that occur during growth. The physiological traits that account for the adaptation include stomata conductance, which acts as the regulator of the photosynthesis process. Plants reduce leaf size during drought to minimize water losses by transpiration and photosynthesis. Sorghum allows accumulation of high chlorophyll content and maintains greenness, which is important for the production of energy. Below is the detailed discussion of the physiological mechanism of sorghum to drought. 

#### 2.1.1. Stay-Green Sorghum

Stay-green is defined as the retention of green leaves under physiological stress, which enhances photosynthesis for production of energy and nutrients in plants. According to [36], there are five types of stay-green that occur in plants where distinct physiological and genetic modifications can be exploited; however, more than one type can be combined. The first type is termed as type A, in which the leaves and stems remain photosynthetically active for a long duration of time. The second is type B, in which leaf senescence occurs slowly at the normal physiological plant growth. The third is type C, which is characterized by acceleration of the production of pigments on the surface of the organ, which lower the expression of senescence. Fourth is type D, which is common to herbs and freezing of vegetables, in which green colour remains with leaf death through freezing and drying. The fifth type is E; it is characterized by high chlorophyll in photosynthetic tissues, which delays the extent of yellowing of leaves and stems similar to type A while maintaining green tissues under limited carbon dioxide fixation [36]. Stay-green in sorghum is an important trait for post-flowering drought tolerance. Stay-green sorghum has the ability to grain-fill under water shortage compared to senescent genotypes [37]. The post-flowering stage is most important for grain filling as one of the important factors of yield determinants [9]. Post-flowering drought has influence on carbon dioxide fixation and translocation as well as transpiration efficiency during water stress; as such, it causes acceleration of leaf senescence and reduces grain yield [36]. Reduced number of tillers and smaller upper leaves contribute to reduced water uptake during pre-flowering, thus keeping water available for plant use at post-flowering drought [38]. Sorghum varieties with low levels of stay-green at pre-flowering and post-flowering stages lack drought-tolerance mechanisms. For instance, leaf senescence starts early in drought sensitive varieties, as leaves lose the ability to synthesize photosynthates, thus causing premature death and lowering grain filling [11]. Drought increases leaf senescence in the plant, especially during the post-flowering stage [7]. Genotypes with drought tolerance show low levels of leaf senescence maintaining green leaves, which accounts for photosynthesis in plants. Sorghum varieties with delayed leaf senescence have high chlorophyll content and chlorophyll content index, and the leaves remain green for a long period of time during post-flowering water stress [39]. Stay-green is the result of a balance between the nitrogen for grain filling during post-flowering and that of vegetative plant growth. During young stages, sorghum plants absorb high levels of nitrogen for vegetative growth that increase high specific-leaf nitrogen during post-flowering drought for production of grains [40]. However, under limited nitrogen content, a high percentage of it will be utilized for grain filling, thus increasing leaves senescence. Deployment of stay-green traits through improvement of delaying leaf senescence is one of the strategies to improve sorghum production in semi-arid areas. It improves grain production, fodder, and quality of sorghum products, with positive correlation to stay-green [5,10]. The critical stage of quantifying the ability of sorghum variety to maintain stay-green is during pre-flowering and post-flowering, when optimum water is needed for vegetative growth and production, respectively. Stay-green sorghum lines are screened in breeding programs to develop drought-tolerant varieties [7]. Stay-green increases tolerance to lodging and has positive correlation with tolerance to stem rot, implying that it remains photo-synthetically active [41,42]. Further studies to identify traits contributing to stay-green expression in sorghum are needed to address drought stress.

#### 2.1.2. Leaf Rolling and Stomata Conductance

Leaf rolling is a mechanism to prevent water loss by reducing leaf surface area during water stress, while stomatal conductance is defined as the measure of the rate of passage of carbon dioxide (CO_2_) coming in and/or outgoing water vapour via the stomata leaf. The positive correlation of leaf rolling against leaf water potential has gained advantage for breeders, who use leaf rolling as one of the parameters for drought-tolerance estimation. Leaf rolling caused by reduction of leaf water potential differs from one plant species to another. Plants with high levels of leaf osmotic adjustment at low leaf water potential have low leaf rolling [43]. Sorghum plants maintain stomata opening under low levels of leaf water potential due to high levels of osmotic adjustment, contributing to drought tolerance in sorghum [44]. In addition, leaf rolling is used for desiccation avoidance, as it has negative influence on transpiration rate caused by changes in leaf stomatal conductance and reduction of leaf area to reduce water loss [45]. Leaf rolling helps to enhance plant survival by stomatal closure. Leaf rolling is a trait used for screening drought-tolerant sorghum lines in the absence of sophisticated instruments for transpiration rate and stomatal closure measurements [43]. Plants with high drought tolerance show low stomatal conductance compared to sensitive plants. Stomatal closure is caused by high leaf temperature and transpiration rate. Plants with high susceptibility to drought are enhanced by high stomatal conductance with low leaf temperature [45].

#### 2.1.3. Chlorophyll Content

Chlorophyll is an important part of the plant that governs the photosynthesis process for the production of food. Chlorophyll is classified into five types: type *a* found in higher plants, *b* in plants and green algae, and chlorophylls *c*, *d,* and *e* in some algae. Chlorophyll, a photosynthetic pigment, is involved in light absorption and plays an important role in plant photosynthesis. Chlorophyll converts sunlight energy to chemical energy in the presence of sunlight and water. Limited sunlight and water reduces chlorophyll production as well as yield in crops. Drought reduces the production of chlorophyll, as it reduces surface area of green leaves and allows high transpiration rate [46]. Drought destroys photosynthetic parts and thus reduces chlorophyll content in plants [47]. Sorghum has the ability to resume photosynthesis and physiological growth after phase out of drought stress, which makes it an important crop in semi-arid areas. Chlorophyll content determines the ability of plants to adapt drought stress [48,49]. High levels of type *a* chlorophyll facilitate chlorophyll content recovery compared to type *b,* which confers drought tolerance in sorghum. Sorghum varieties that are drought tolerant might contain higher ratios of type *a* chlorophyll than type *b,* which is useful for introgression into the recipient parents for improvement of drought tolerance [23].

### 2.2. Morphological Mechanism

The morphological traits related to drought are one of the important aspects to look for when screening the promising sorghum lines for drought tolerance [12]. Morphological responses to drought in sorghum involve traits that are associated with drought tolerance, for instance, plant height, leaf arrangement, roots and root system, root to shoot ratio, and biomass [50]. Plants with large weight of root biomass production have a high chance to tolerate water stress [51]. The biomass production depends on roots and shoots growth in plants. Drought affects more shoot growth than root growth in sorghum due to high level of temperature, which increases rate of transpiration in plants [52].

#### 2.2.1. Roots and Root System

Root architecture and root branching influence drought tolerance, as they help to reserve water during water stress [53]. Roots grow about 2 to 3 cm per day and are the first parts of the plant affected by drought stress. The rate of root growth is high in the soil at the booting stage; this enables uptake of water at a distance of 1.6 m lateral from the plant for drought-tolerant plants [54,55]. Sorghum genotypes with seminal roots and large vessel diameters have high tolerance in limited water environments. Root distribution and root system structure in sorghum depends on the carbon partitioning to the roots, which increases plant survival during drought stress [56]. Plants with high ability to establish high root growth in presence of water have high ability to tolerate water deficit due to increased root growth, which increases contact with soil, thereby enhancing water uptake [57]. During drought conditions, the plant utilizes water available in the root zone, while crops with high tolerance to drought absorb water from deep soil to survive [31]. Studies have shown that drought-tolerant crop species have deeper roots and longer lateral root systems [58,59]. In addition, thick roots and large branches of roots penetrate the compact soil, which facilitates high uptake of water for plant use [60]. Therefore, the association of root architecture and root branching should be exploited to improve drought tolerance in sorghum, as it helps to reserve water during water stress.

#### 2.2.2. Biomass Production

Biomass refers to the production of dry matter after extraction of water by drying in the oven. Biomass production differs among plant species and varieties. It is among the traits that are used as an indicator of drought tolerance in plants, including sorghum. Drought-tolerant lines are characterized by high production of biomass as the way of adaptation for survival. About 47% biomass of sorghum is produced at post-flowering during water stress [40]. Biomass is grouped into two types: the first type is above-ground biomass, which consists of dry matter of plant parts above the ground, for instance, shoot. The second type is below-ground biomass, composed of dry matter below the ground, for instance, roots. Biomass accumulation in sorghum is contributed by high efficiency of photosynthesis, which is driven by high chlorophyll content in leaves, stay-green, and high concentration of carbon-dioxide (CO_2_) in the atmosphere [61,62]. Borrell et al. [7] confirmed that biomass accumulation in sorghum is correlated with stay-green; sorghum genotypes with high stay-green have high biomass accumulation compared to non-stay-green genotypes. High production of biomass at the post-flowering growth stage increases yield potential more than partitioning [8]. Increase of biomass accumulation in sorghum could be due to enzymes that are active during moisture stress to rescue plants from wilting. Therefore, it is recommended to take into consideration biomass production when testing lines for drought tolerance in sorghum.

#### 2.2.3. Root to Shoot Ratio

The production of root and shoot depends on availability of water for plant intake. Limited water affects root and shoot development in plants. In sorghum, the root to shoot ratio increases with decrease of soil moisture as the mechanism of survival [63]. However, there is a variation in efficiency for maintaining root to shoot ratio among sorghum varieties. Sorghum with a high level of stay-green accumulates a large weight of biomass to support the plant [7]. The primary root growth of sorghum is slower than secondary root growing from the root crown. The strength of the root system and biomass production therefore depend mainly on the secondary growth root system [64]. Root morphology, root biomass, and structures of root systems enhance tolerance of sorghum in the limited soil moisture [65]. Thus, further studies to understand the mechanism that confers increase of root to shoot ratio during drought stress should be given priority in sorghum improvement.

### 2.3. Biochemical Mechanism

Biochemical mechanisms involve the biochemical traits, such as proline and photosynthetic pigments, which assist plant to adapt drought conditions [51]. Drought stress triggers the production of reaction oxygen species and breakdown of the cellular membrane, which hinders the metabolic reactions in plants. Studies have shown that drought-sensitive crops, like maize, barley, and tobacco, accumulate higher H_2_O_2_ and lipid peroxidation than drought-tolerant crops [66]. Drought stresses enhance the reaction of enzyme antioxidants; it has been reported that plants that produce more antioxidants have more chance of tolerating drought than those producing less antioxidants [67]. These findings indicate that activities of those enzymes increase in drought-stress-tolerant sorghum [68]. Such findings point out the need to further study the mechanism of antioxidant enzymes systems in relation to drought stress in sorghum. Sorghum accumulates high content of proline as the means of minimizing drought effects [51]. In addition, nitrogen also has a significant contribution to drought tolerance in sorghum. Drought induces biochemical reactions in plants, which persuades the secretion of compatible solutes, dehydrins, and drought-inducible proteins. The compatible solutes are responsible for covering plants from high levels of osmotic stress during water stress [69]. The accumulation of compatible solutes in plants is the indicator of drought stress. The production of solutes plays the mechanism for adaptation of drought conditions. Biochemicals are accumulated in herbaceous plants as the means of plant protection, and such solutes should be exploited for improvement of crops [70]. Heat Shock Proteins (HSPs) and dehydrins (DHNs) are classifications of proteins that increase with increase of drought stress in sorghum and maize [70]. Thus, traits related to biochemical mechanisms for drought tolerance in sorghum need to be studied at molecular level to widen the chance of detecting QTLs related with drought tolerance in the biochemical compounds.

## 3. Marker-Assisted Selection for Enhancing Drought Tolerance in Sorghum

### 3.1. Molecular Markers in Sorghum Genotyping

Molecular markers help to select the parents with contrasting traits as the basis for improvement of new traits of target [71]. With high variation present between and within hybrid and local accessions, characterization is important to identify suitable genotypes for use in agriculture and preserving in the gene bank for future use [72]. Morphological markers are used for analyses of genetic diversity; however, it is difficult to map morphological markers [73]. Morphological features are influenced by environmental factors [74]. Isozymes have been used, but their polymorphism does not provide sufficient information for diversity studies in sorghum [75]. The quality of molecular markers for the identification of closeness and distant relationships among accessions has been varying. For instance, restriction fragment length polymorphism (RFLP), amplified fragment length polymorphism (AFLP) [76], and Simple Sequence Repeat (SSR) markers have polymorphism with high polymorphic information content (PIC) and Shannon diversity index. Random amplified polymorphic DNA (RAPD) markers are simple, time-saving due to their rapidity, and require small amounts of DNA [77]. The advantages of SSR are that they are co-dominant, polymorphic, highly informative, easy to apply with relative high resolution, and less expensive for molecular biotechnology studies [78]. Among the number of molecular markers for sorghum genotyping, single-nucleotide polymorphisms (SNP) markers are currently at the peak due to high abundance and can accommodate the whole genome with high throughput and high resolution compared to others. SNP markers have the ability to identify the diversity to a single base level [79]. SNPs markers can indicate the variability of a single nucleotide in the DNA present in the genome. Due to high efficiency, the number of SNPs and variations available in all regions of the plant genome are detected [80]. SNPs markers can be exploited from genomic or from the expressed sequence tag sequences by high-throughput sequencing technology and can be obtained in the PCR products [81].

### 3.2. Maker-Assisted Backcrossing

**Figure 2:**
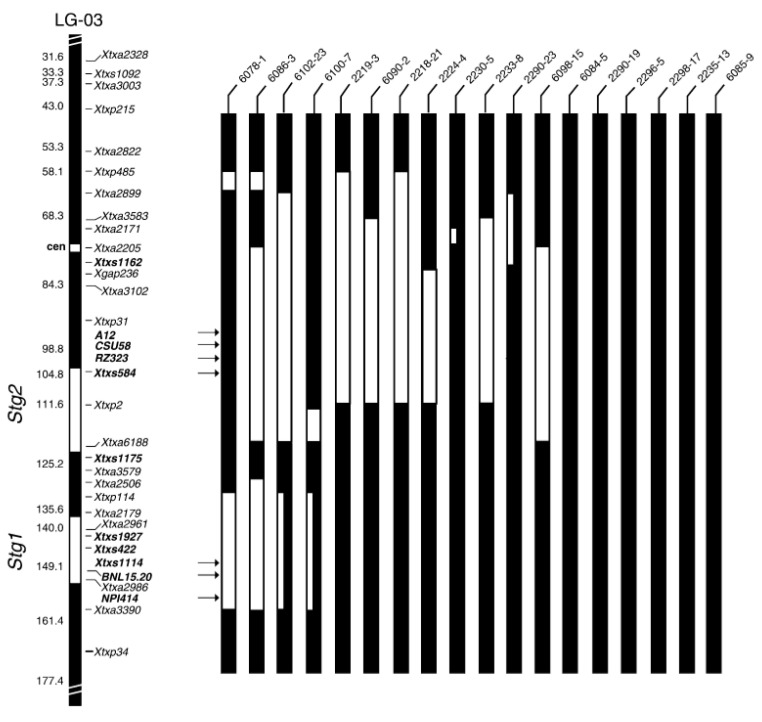
Linkage group of *Stg1* and *Stg2* in SB1-03 [86].

**Figure 3:**
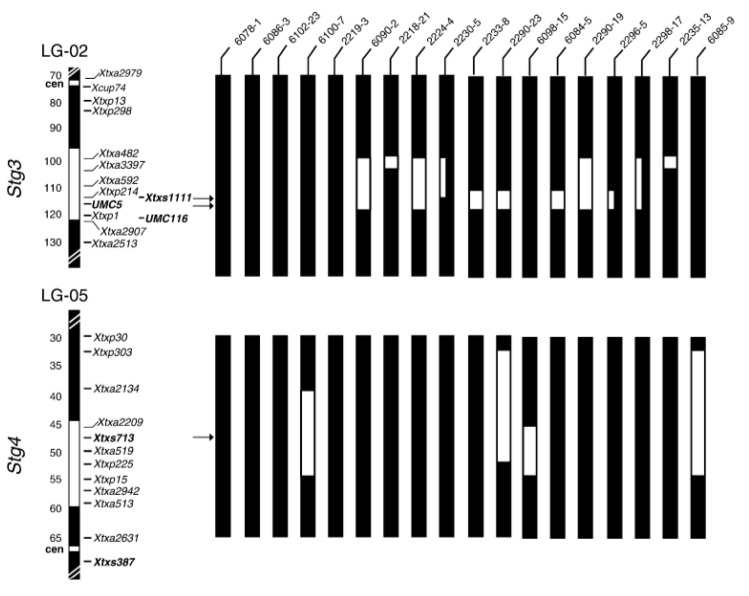
Linkage group of *Stg3* and *Stg4* in SBI-02 and SB1-05 chromosomes [86].

Backcrossing refers to the transfer of alleles at one or more loci from the donor parent to the elite recurrent parent [82]. Backcrossing is tedious and time consuming; the innovation of molecular markers has been useful, as it helps to identify QTLs associated with the expression of traits of interest. For instance, various markers (simple sequence repeat and single nucleotide polymorphism) have been selected for mapping QTLs of stay-green in sorghum. These markers simplify the breeding programs by reducing the breeding cycles, thus shortening the duration of releasing new, improved varieties. The introgression of alleles of the elite trait is attained after six backcrosses, with genome recovery of recurrent parents by 99.2% [82]. Theoretically, the recurrent parent genome is recovered by half of each backcross; for instance, two backcrosses are recovered by 87.5%. Nonetheless, practically, the recovery of recurrent parents may exceed or maybe be reduced. Plant breeders use marker-assisted backcrossing as the tool for selection of the best trait that contains alleles with high recovery of recurrent parent genome. Most traits of interest in the field crops have been difficult to exploit through conventional breeding [83]. The use of marker-assisted backcrossing can exploit traits from one variety to another in a number of crops [84]. This approach helps to identify tightly linked QTLs associated with traits of interest. They are transferred to the potential sorghum lines through marker-assisted backcrossing, thus reducing the breeding cycle compared to a conventional approach. It helps to phenotype large populations with consistency across the environment; however, QTLs are influenced by variation in environmental factors [84]. Testing the effect of water stress to sorghum across environments is important. Researchers have concentrated on developing new varieties that are drought tolerant because of climate change occurring in most parts of the world. For sorghum, a number of molecular markers have been utilized to identify the QTLs associated with stay-green, like delayed leaf senescence, leaf rolling, chlorophyll content, water-use efficiency, and yield [42,85,86]. Figure 2 shows the linkage group of the *Stg1* and *Stg2* QTLs, and Figure 3 shows *Stg3* and *Stg4.* These QTLs are tightly linked to the genes expressing post-flowering drought tolerance and yield, which are used for backcrossing to senescence sorghum varieties for improvement of drought tolerance [20]. The four *Stg* QTLs together contribute 53.5% of phenotypic variance in the “B35” × “Tx7000” RIL population. The variability simplifies screening of stay-green traits for improvement of post-flowering drought tolerance [87].

QTLs are mapped across the environments, while others are specific to some environments [88]. To achieve exploitation of QTLs from the donor parents to recurrent parents, a large number of polymorphic, high-resolution, high-throughput, co-dominant, and informative molecular markers, such as SSRs and SNPs, are recommended for backcrossing [89]. For instance, SSRs markers have mapped *Stg1*–*Stg4* QTLs that account for 10–30% phenotypic variance of drought tolerance in sorghum (Table 1). Recently, ICRISAT mapped *Stg3A* and *Stg3B* QTLs in chromosomes using SNPs markers in sorghum. Those QTLs have high efficiency for delaying leaf senescence during post-flowering drought stress. Mwamahonje et al. [90] introduced *Stg3A* and *Stg3B* QTLs into farmers’ preferred sorghum varieties in Tanzania. The QTLs were successfully introgressed by snpSB00049, snpSB00053, and snpSB00054 SNPs markers for *Stg3A* QTL and snpSB00101, snpSB00102, and snpSB00103 SNPs markers for *Stg3B* QTL, which accounted 30% of grain yield increase and stay-green in the water-stressed environments. However, some of the markers failed to detect QTLs, indicating needs of further mapping of *Stg3A* and *Stg3B* to diversify relevant markers for drought-tolerance improvement.

**Figure 4:**
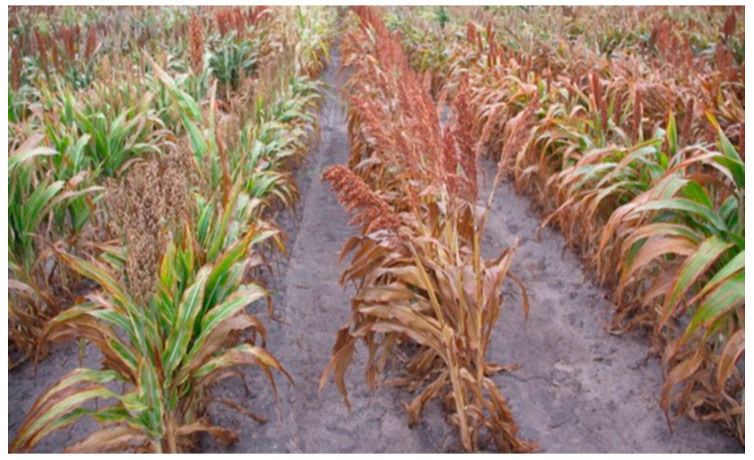
Expression of introgressed stay-green QTLs lines left and unintrogessed lines of sorghum, right, Photo—2019.

Therefore, molecular markers have been useful for identification of genes associated with complex drought tolerance due to small genome size of sorghum [94]. By using molecular markers, a number of sorghum linkage maps associated with drought tolerance have been identified [95]. Marker-assisted selection is useful, as it can exploit traits of interest easily compared to phenotyping [9]. Molecular markers for donor and recurrent parents play a major role for elimination of linkage drag, enhancing the recovery of a recurrent parent’s genome. *Stg1, Stg2, Stg3,* and *Stg4* QTLs originated from donor parent B35 are stable and used for introgression in several genetic backgrounds, with senescence lines through marker-assisted backcrossing [7,20,39]. The phenotypic expression of stay-green on introgression sorghum lines have been screened under field conditions in reference to genotypic data. Harris-Shultz et al. [96] proved the role of marker-assisted backcrossing on the phenotypic expression of introgressed lines of sorghum crop, as in Figure 4 which shows expression of stay-green in the introgressed line during drought. This information is used as the basis for further mapping of stay-green QTLs that can be used for introgression to other varieties for sustainable sorghum production. Furthermore, epistatic interactions within and between stay-green loci have been reported in the sorghum genome; these interactions within the phenotypes can be exploited by the use of Near-isogenic lines (NILs) and applied in the sorghum breeding program. Marker-assisted backcrossing has been applied in cereal crops, including sorghum. Marker-assisted backcrossing remains among the important tools for introgression of traits of interest into the lines that lack to diversify the improvement of new traits that are demand driven.

### 3.3. Foreground and Background Selection

Foreground selection refers to selection of samples with molecular makers of the donor parent with particular target of locus. The aim of foreground selection makers is to maintain the locus of target to heterozygous state of both parents up to final backcrosses [97]. Foreground selection is achieved by marker-assisted backcrossing, where traits of interest are introgressed from donor parents to the recurrent parents for improvement. Some of the traits that have been useful for introgression into recurrent parents through this approach include drought tolerance, high yielding, shoot fly resistance, and *Striga* species resistance in sorghum. Foreground selection markers mSBCIR238 *Stg3*, Xtxp72 *StgB*, mSBCIR222 *Stg2*, mSBCIR314 *Stg1* and *2*, Xtxp225 *Stg4,* and Xtxp285 *Stg1* were mapped for drought-tolerance enhancement by backcrossing donor parent B35 line with recurrent parents [98]. SSR makers mapped five QTLs for *Striga* resistance in chromosome A /linkage group 1, chromosome J1/ linkage group 5, chromosome B /linkage group 2, chromosome I /linkage group 6, and chromosome J2/ linkage group 5 [99,100]. High yield is a complex trait contributed by several QTLs in chromosome 1, while sorghum flowering QTLs are located in chromosome 2 [101]. These QTLs together significantly contribute to enhancing grain yield in sorghum. On the other hand, the essence of background selection is to reduce donor parent alleles to remain with the desired target gene. Background selection occurs when traits of a recurrent parent is recovered while receiving new specific traits from the donor parent for enhancement [97]. It aims to minimize the size of the introgressed region to remain with the gene of target following selection of non-deleterious loci of particular traits during backcrossing. It is achieved by identifying the best recombinants between the genes of target that are closely linked to the markers [102]. Edema and Amoding, [20] in their study on background selection markers in F1 lines, found that Xcup62, Xcup73a, and Xtxp208a markers were located in linkage group A. Gpsb014a, Xtxp286, Xtxp304, mSBCIR223, and Xtxp050 markers were located in linkage group B, and Xcup32 and Xtxp033a were located in linkage group C, after crossing donor parentB35 versus recurrent parent Seredo using SSR markers. Single-nucleotide polymorphism markers could work better to detect linkage groups due to higher efficiency compared to SSR markers.

### 3.4. QTL Mapping for Yield in Sorghum

QTL mapping is the exploitation of QTLs associated with genes coding for expression of traits of interest using molecular markers. For instance, the yield-related components in sorghum are conferred by a number of genes [103]. These genes are associated with QTLs, which express agronomic-related traits, such as yield components and abiotic stress tolerance. These QTLs are traced using molecular markers. Molecular markers are useful for mapping of QTL located close to the genes expressing the traits. The markers that are successful and lead to identification of QTL of agronomic traits of importance are recorded as the markers of that QTL [104]. Marker-assisted selection has a role to play for the improvement of sorghum yield. The exploitation of QTLs for high yield in sorghum involves mapping of QTLs using several DNA markers [105]. During optimization, only markers that are tightly linked with candidate QTLs for high yield are screened for sorghum improvement by marker-assisted selection [105]. The study of genetic diversity focusing on characteristics of drought-tolerant, yield, and yield-related traits, such as plant height, number of leaves, and root biomass, is important to increase heritability and genetic gain for sorghum improvement. A number of QTLs for yield and yield-related traits have been exploited for sorghum improvement. For instance, PSTOL1 and Sb07g02840 genes are associated with increase of root diameter, which enhances sorghum grain yield. The QTLs (Gy-3, SA2–3 located in sorghum chromosome 3 (SBI-03); Gy/SA2–3, at position ~71Mb in SBI-03; and Gy/RD-7 QTL at 3.6Mb in SBI-07) are linked to yield-related traits, such as root morphology and surface area of fine roots, which help to increase yield in sorghum [106]. In addition, Reddy et al. [92] found three QTLs for sorghum yield in SBI-09, one in SBI-04, and one in SBI-06. At least one QTL for panicle was detected on SBI-09, SBI-04, and SBI-06. QGy-dsr06-1 is the major QTLs of panicle weight in sorghum, contributing 11.4% of phenotypic variance [92]. QTLqYLD1.1 in SBI-01 enhances sorghum grain yield during water stress and moisture conditions. Such QTLs should be incorporated to the sorghum lines that lack them for sorghum yield improvement. Yield trait is controlled by several QTLs available in every chromosome in sorghum [23,105,107]. Of the QTLs that have been exploited to enhance sorghum yield, 20% of them are allocated in SBI-01. This conclusion calls upon further research to explore new QTLs that are more useful for improvement of yield than the current QTLs. Plant height, number of tillers, panicle weight, grain weight, and stay-green are collated with grain yield in sorghum [10]. Nevertheless, further studies on mapping QTLs for yield and yield components require more attention to exploit specific QTLs that are highly close to the gene coding for yield to maximize harvesting. Several studies have reported average contribution QTLs in sorghum yield and call upon further mapping of QTLs, which will contribute yield above average of the current reported researches [91,94,108].

### 3.5. QTL Pyramiding for Drought Tolerance in Sorghum

QTL pyramiding involves crossing of one NIL to another, composing different, useful QTLs by subsequent marker-assisted selection to produce new lines with both advantageous traits. With gradual increase of temperature and drought, especially in semi-arid areas, pyramiding QTLs for drought tolerant and yield is recommended, though pyramiding technique is tedious and time consuming [109]. The transfer of traits of interest from the donor parents to the recipient is recommended for the same species [110]. QTL pyramiding improves drought tolerance in sorghum; however, the performance in a well-watered environment yields higher than in the water-stressed environment. For instance, introgression lines with *Stg3* and *Stg1+ 2* reduce yield by 10% in water-stressed conditions compared with well-watered conditions, indicating higher tolerance compared to other *Stgs,* which indicate 18–23% as moderate tolerance in sorghum [111]. However, studies report that crude protein, total soluble carbohydrate, and proline contents increase under drought stress conditions; the increase of these traits have contributed to an increase of quality grain nutrition that improves human health [12]. Pyramiding involves combination of QTLs with different efficiency to enhance the expression of stay-green traits [42]. Use of molecular markers helps to detect QTLs that can be pyramided to improve drought tolerance in sorghum [92]. Pyramiding of QTLs may fail to improve stay-green in sorghum due to incompatible gene action, which does not allow expression of intended traits [39]. The success of pyramiding is subject to genetic architecture and correct mapping of QTL of the trait [111]. During QTLs pyramiding, only the best expression of the trait of interest is selected for further evaluation in multi-location trials before approval as new varieties. Studies have suggested that mapping of QTLs for expression of stay-green using molecular markers are important for pyramiding to enhance heritability of new lines [80]. Every QTL contributes a small percent to enable expression of the stay-green trait, which needs pyramiding to assemble QTLs for expression [39,42]. Pyramiding of stay-green QTLs during pre-flowering and post-flowering drought in sorghum increases chance of developing new, promising lines with valued traits by marker-assisted backcrossing [9,97]. Other traits that have been suggested for exploitation by pyramiding methods are *Striga* resistance [21], high yield, and plant height. These findings assist plant breeders to map the potential QTLs of traits that are introgressed and can be expressed after pyramiding [112]. To achieve pyramiding, efficient molecular markers for mapping QTLs need to be exploited to facilitate molecular breeding programs in sorghum. In pyramiding study, only pyramided QTLs that show expression of traits are recommended for selection in sorghum improvement.

## 4. Conclusions

Drought is a complex trait that is controlled by several genes in crops that makes it difficult to screen the best lines. It needs different selection tools for screening drought tolerance lines that cope with drought stress. The integration of marker-assisted selection plays the major role to enhance screening and mapping for stay-green QTL of the promising sorghum lines. Stay-green in sorghum should be exploited for backcrossing to drought-stress-sensitive lines to strengthen the tolerance. The optimization of current markers with high efficiency to detect QTL for stay-green in sorghum is imperative. There is a need to continue mapping QTL associated with genes coding for stay-green of above-ground and underground and root biomass to supplement information for mechanism of drought tolerance in sorghum. Such information will be used as the base for sorghum breeders to screen the promising sorghum lines that can tolerate drought and yield high. Therefore, sorghum varieties that have robust mechanisms of tolerance to drought should be given priority in research, especially in semi-arid areas, to sustain food security and household economy. Local and improved sorghum varieties that respond well to drought should be maintained in the genebank by sorghum breeders for further breeding programs as the adaptation strategy to drought now and in future.

## Figures and Tables

**Table 1:** Molecular Markers and Stay-green QTLs for Drought Tolerance in Sorghum.

Molecular Marker	QTL	Position in Chromosome	PV (%)	Reference
Xtxp114, Xtxp38, xiabxp 3 7 8, SSR markers	*Stg1*	SB1-03	20	[87,91]
XnhsbSFCILP67, Xtxp120, Xtxs584, and Xtxp31, SSR markers	*Stg2*	SB1-03	30	[87]
Xtxs1307, Xtxs1111, Xtxp1, Xtxp56, Xtxp286, SSRs markers	*Stg3*	SB1-02	16	[42,86]
Xtxs713, Xtxs387, Xtxp225, Xtxp15, SSR markers	*Stg4*	SB1-05	10	[42,86,92]
snpSB00049, snpSB00053, and snpSB00054, SNPs markers	*Stg3A*	SB1-02	31	[90,93]
snpSB00101, snpSB00102, and snpSB00103, SNPs markers	*Stg3B*	SB1-02	31	[90,93]

## Data Availability

Not applicable.
